# Federated deep learning for detecting COVID-19 lung abnormalities in CT: a privacy-preserving multinational validation study

**DOI:** 10.1038/s41746-021-00431-6

**Published:** 2021-03-29

**Authors:** Qi Dou, Tiffany Y. So, Meirui Jiang, Quande Liu, Varut Vardhanabhuti, Georgios Kaissis, Zeju Li, Weixin Si, Heather H. C. Lee, Kevin Yu, Zuxin Feng, Li Dong, Egon Burian, Friederike Jungmann, Rickmer Braren, Marcus Makowski, Bernhard Kainz, Daniel Rueckert, Ben Glocker, Simon C. H. Yu, Pheng Ann Heng

**Affiliations:** 1 Department of Computer Science and Engineering, The Chinese University of Hong Kong, Hong Kong SAR, China; 2 Department of Imaging and Interventional Radiology, The Chinese University of Hong Kong, Hong Kong SAR, China; 3 Department of Diagnostic Radiology, Li Ka Shing Faculty of Medicine, The University of Hong Kong, Hong Kong SAR, China; 4 Biomedical Image Analysis Group, Imperial College London, London, UK; 5 Institute for Diagnostic and Interventional Radiology, Technical University of Munich, School of Medicine, Munich, Germany; 6 OpenMined, Oxford, UK; 7 Shenzhen Institutes of Advanced Technology, Chinese Academy of Sciences, Shenzhen, Guangdong China; 8 Department of Diagnostic Radiology, Princess Margaret Hospital, Hong Kong SAR, China; 9 Department of Radiology, Tuen Muen Hospital, Hong Kong SAR, China; 10 Department of Emergency Medicine, Peking University ShenZhen Hospital, Shenzhen, Guangdong China; 11 Department of Radiology, Zhijiang People’s Hospital, Zhijiang, Hubei China; 12 German Cancer Research Center (DKFZ), Heidelberg, Germany; 13 AI in Medicine and Healthcare, Technical University of Munich, School of Informatics and Medicine, Munich, Germany

**Keywords:** Diagnostic markers, Computed tomography

## Abstract

Data privacy mechanisms are essential for rapidly scaling medical training databases to capture the heterogeneity of patient data distributions toward robust and generalizable machine learning systems. In the current COVID-19 pandemic, a major focus of artificial intelligence (AI) is interpreting chest CT, which can be readily used in the assessment and management of the disease. This paper demonstrates the feasibility of a federated learning method for detecting COVID-19 related CT abnormalities with external validation on patients from a multinational study. We recruited 132 patients from seven multinational different centers, with three internal hospitals from Hong Kong for training and testing, and four external, independent datasets from Mainland China and Germany, for validating model generalizability. We also conducted case studies on longitudinal scans for automated estimation of lesion burden for hospitalized COVID-19 patients. We explore the federated learning algorithms to develop a privacy-preserving AI model for COVID-19 medical image diagnosis with good generalization capability on unseen multinational datasets. Federated learning could provide an effective mechanism during pandemics to rapidly develop clinically useful AI across institutions and countries overcoming the burden of central aggregation of large amounts of sensitive data.

## Introduction

The COVID-19 outbreak, caused by the novel coronavirus SARS-CoV-2, has presented a public health crisis worldwide. According to data compiled by the Center for Systems Science and Engineering at Johns Hopkins University^1^, the global number of COVID-19 cases exceeded 64.69 million with over 1.49 million total deaths as of 5 December 2020, and the pandemic continues to spread or recur across continents especially in low-income countries. At the peak of the pandemic in early 2020, the clinical capacities to respond were overloaded in several countries, even with advanced healthcare systems such as present in Italy. Moreover, existing digital healthcare systems were rapidly overwhelmed and frontline clinicians challenged with an unprecedented amount of emergency workload for data analysis in a hitherto unseen disease entity^2^. Artificial intelligence (AI) has the potential to provide access to accurate, low-cost, and scalable solutions in combating COVID-19 through automated analysis of patient data.

Multicenter collaborative research efforts have been expected to coordinate data sources for maximizing the potential of data-driven AI technologies^3,4^. To this end, both the training and testing aspects should be considered with equal importance for data and model sharing. For the training phase, aggregating multiple data sources helps improve model robustness and generalizability, because scaling amounts of data with various imaging protocols and diverse patient populations could help reduce model bias^5^. Enabling privacy protected data sharing across clinical centers is advocated as an essential pathway to promote collaborations internationally yet underexplored so far^6,7^. For the testing phase, validation of AI models on multiple, unseen, independent external cohorts has to be a crucial criteria for assessing scalable usability toward wide model sharing^8,9^. Recent study^10^ has revealed the potential of federated learning models for generalizability outside federation on brain tumor application. However, there is still little evidence been reported to date on the generalization performance of decentrally developed AI models for widely collected COVID-19 cohorts, especially in the setting of multinational evaluation of heterogeneous patient cohorts.

A major focus of AI fighting against COVID-19 is interpreting radiological images, mainly chest CT which has been widely applied for detecting lung changes to inform patient management, assessment of severity, and monitoring of the disease^11–13^. The main findings of COVID-19 infection on CT scans are bilateral and peripheral ground-glass and consolidative pulmonary opacities^14^. These are currently clinically interpreted in a qualitative manner, but having a method that can quantitatively measure the disease burden and changes over time will be valuable for patient surveillance. Existing AI models to date are mostly designed for lung lesion segmentation using convolutional neural networks (CNNs)^15,16^, requiring dense pixel-wise labels through time-consuming, labor-intensive manual annotation from experts who are scarce during the crisis. We instead consider detecting lesion bounding boxes, for which annotations are easier and quicker to obtain while maintaining clinical utility of quantifying the burden of infection.

We aimed to demonstrate the feasibility of training a deep CNN-based AI model for automated detection of lesions from COVID-19 CT images, using a privacy-preserving method which does not require exchange of data between centers nor data to be stored centrally. Model validations were conducted using local and external datasets (including one international cohort) with comparison to expert radiologists’ interpretations. In addition, case studies with longitudinal scans were also performed for automated estimation of the lesion progression to support monitoring hospitalized patients.

This study explores the potential of federated learning methods to develop a privacy-preserving AI system for the real-world problem of automated COVID-19 image interpretation. A CNN-based model has been successfully trained on decentralized multicenter data to detect lesions from COVID-19 CT images, with wide generalizability to external patients (from Germany, China, and one publicly available dataset). These attributes showed the potential of federated learning to build generalizable, low-cost, and scalable AI tools for image-based disease diagnosis and management, both for research and clinical care.

## Results

### Study design and participants

In this multicenter study, the internal datasets were collected from three local hospitals in Hong Kong, i.e., Prince Wales Hospital (Internal-Set-1: PWH), Princess Margaret Hospital (Internal-Set-2: PMH), and Tuen Mun Hospital (Internal-Set-3: TMH). A total of 75 patients (mean age: 47.1 ± 17.5, 32 female and 43 male) with confirmed COVID-19 infection (positive RT-PCR tests) were enrolled in this study. These retrospective CT images were collected during the time period from 24 Jan 2020 to 16 Apr 2020. The ethical approvals were obtained in accordance with all relevant laws and regulations for each recruiting hospital (see details in supplementary p. 2). Waiver of informed consent was obtained by the Ethics Commission of the designated hospitals. The regions of ground-glass opacification and consolidation, which are the two main signs of COVID-19 assessed on CT images, were manually annotated with bounding boxes (see detailed annotation process in supplementary p. 2).

To evaluate the robustness and generalizability of our AI model beyond local centers to wider data distributions of imaging protocols and patient populations, we used four datasets outside Hong Kong for external validation: (1) External-Set-1: a publicly released COVID-19 CT dataset (https://coronacases.org/) of 10 patients originally collected by Wenzhou Medical University, China, with a third-party lesion annotation released by Ma et al.^17^; (2) External-Set-2: a private dataset of 35 patients (collected during 02 Feb 2020 to 30 Mar 2020) from BioMedIA research group collected at the Klinikum rechts der Isar, Technical University of Munich, Germany, with lesions independently labeled locally; (3) External-Set-3: a private dataset of 10 patients (collected during 25 Jan 2020 to 04 Mar 2020) from Peking University Shenzhen Hospital, China, with lesions independently labeled locally; (4) External-Set-4: a private dataset with longitudinal studies of two patients (collected during 23 Jan 2020 to 19 Mar 2020) from Zhijiang People’s Hospital in Hubei, China, with hospitalized records acquired. All these included patients had confirmed COVID-19 infection with positive RT-PCR tests. Each participating external private center obtained individual ethical approval in accordance with respective relevant laws and regulations. The inference codes and AI models were sent to each center for independent held-out testing as external validations.

Table 1 lists the demographic variables and imaging protocols of the recruited seven centers including three internal cohorts from Hong Kong, and four external cohorts from Mainland China and Germany. The real-world heterogeneous environments of CT medical imaging in clinical practice could be reflected to a certain extent in this proof-of-concept study.

**Table 1:** The demographic variables and imaging protocols of the recruited seven centers including internal cohorts from Hong Kong, and external cohorts from Mainland China and Germany.

Cohorts		Internal- Set-1	Internal- Set-2	Internal- Set-3	External- Set-1	External- Set-2	External- Set-3	External- Set-4
Demographic variables	Number	7	65	3	10	35	10	2
	Female	5	25	2	4	13	7	0
	Male	2	40	1	6	22	3	2
	Age (years)	32.7 (15–59)	47.7 (12–80)	67.0 (51–91)	47.1 (32–64)	60.9 (27–88)	54.5 (16–70)	60.0 (57–63)
Imaging protocols	Axial resolution (mm)	0.64 (0.63–0.7)	0.64 (0.51–0.78)	0.69 (0.66–0.75)	0.72 (0.68–0.81)	0.73 (0.57–0.91)	0.71 (0.64–0.78)	0.73 (0.66–0.79)
	Slice thickness (mm)	0.625	1.25	0.7	1.0 (7 cases) 1.5 (3 cases)	3.0	0.625 (4 cases) 1.0 (5 cases) 1.25 (1 case)	1.25
	Manufactor	GE Medical Systems; Milwaukee, WI, USA	GE Medical Systems; Milwaukee, WI, USA	SIEMENS Healthineers; Forchheim, Germany	n/a	Philips Healthcare, Cleveland, OH, USA	TOSHIBA, Canon Medical Systems, Tochigi, Japan/Anke High-tech Co., Ltd., Shenzhen, China	GE Medical Systems; Milwaukee, WI, United States
	Manufacturer’s Model	LightSpeed VCT	LightSpeed Pro 32	SOMATOM Definition AS+	n/a	iCT 256	Aquilion/ANATOM 64 Precision	Optima CT680 Series
	Tube voltage (kvp)	120	120	120	n/a	120	120	120
	Tube current (mA)	120–200	50–502	229–399	n/a	30–347	180–350	209–401
	Patient position	Feet First-Supine	Feet First-Supine	Feet First-Supine	n/a	Head First-Supine	Head First -Supine	Head First-Supine
	Intravenous contrast	Non-contrast	Non-contrast	Non-contrast	Non-contrast	Non-contrast	Non-contrast	Non-contrast
	Inspiration	Inspiratory	Inspiratory	Inspiratory	Inspiratory	Inspiratory	Inspiratory	Inspiratory

The numbers (including age, axial resolution) are presented as average (range). In imaging protocol, the slice thickness is presented as a specific number (number of cases), and the tube current is presented in range. The n/a stands for that the protocol parameters are missing from the anonymized public cohort.

### Experimental settings

**Fig. 1:**
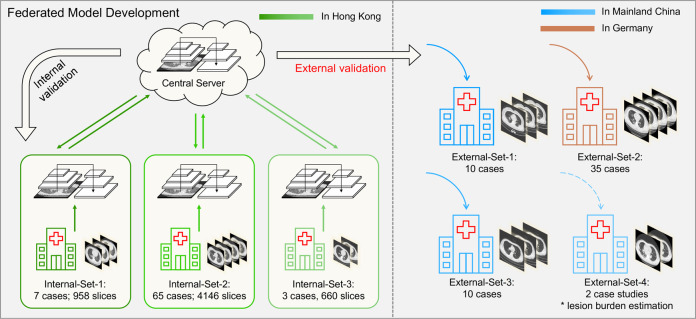
Overview of our AI scheme to develop a privacy-preserving CNN-based model for detecting CT abnormalities in COVID-19 patients with a multinational validation study. A privacy-preserving AI system was developed with CT data from three hospitals in Hong Kong using federated learning, and then the generalizability was validated on external cohorts from Mainland China and Germany.

An overview of our study scheme is illustrated in Fig. 1. We developed our CNN-based deep learning model for CT lesion detection using the three internal datasets with federated learning. Transfer learning was used leveraging our previously developed detection model^18^ on the large-scale public DeepLesion dataset^19^ (see details in the “Methods” section). The network training was conducted on the training subsets. Details of annotated CT lesion datasets and the random subset splits are listed in Table 2. For validation, the established models were first evaluated on the internal testing subsets. To further study how the AI models would generalize to completely unseen centers and patient cohorts, we conducted external validations on the External-Set-1, External-Set-2, and External-Set-3. In addition, to explore the potential usefulness of AI tools for monitoring the change of lesion burden for hospitalized patients, we also performed external validation with External-Set-4, using two case studies with sequential CT scans over time. Note that the data from all four external centers were used solely for testing purposes.

**Table 2:** Summary of annotated lesions and data splits for AI model training and testing.

Cohorts	Internal-Set-1	Internal-Set-2	Internal-Set-3	External-Set-1	External-Set-2	External-Set-3
Volume number	7	65	3	10	35	10
Training split	5	53	2	–	–	–
Testing split	2	12	1	10	35	10
Lesion slice numbers	958	4146	660	1200	7199	1576
Training split	676	3333	521	–	–	–
Testing split	282	813	139	1200	7199	1576
Lesion numbers	1566	10,544	1985	2655	21,539	3677
Training split	1117	9398	1831	–	–	–
Testing split	449	1146	154	2655	21539	3677

The table lists the number of CT volumes, number of slices containing lesions, and number of individual lesion regions for all the manually annotated sets. For internal cohorts, the data split for training and testing are presented correspondingly in these three aspects. For external cohorts, the data were all used for testing.

To analyze the benefit of scaling the amount of training data through multicenter learning, we also established baseline settings to train the models for comparisons. For each internal site, we trained an individual model with standard single-site training, obtaining three independent networks, denoted with *Individual-model-1* (trained on Internal-Set-1), *Individual-model-2* (trained on Internal-Set-2), and *Individual-model-3* (trained on Internal-Set-3). In addition, we established the comparison method of *model ensemble* of these three individual models, i.e., running each individual model on the testing cohorts and merging their prediction results. We have also added a baseline of training a single *joint model* with all data centralized. For all these five comparison settings, both internal and external validations were conducted following the same evaluation scheme as the *federated learning model*.

Overall, we conducted experiments representing six settings, i.e., three single-center models, their ensemble, a joint model, and a federated learning model. Besides the joint model, all other five methods were free from data exchange or centralization in the multicenter study setting, thus protecting the privacy of the patient health data.

### Statistical analysis

This study aimed to analyze the effectiveness of federated deep learning on the task of chest CT abnormality detection for COVID-19 with evaluating the performance of AI models trained from multicenter data and validated on both the internal and external testing datasets. The evaluation was conducted based on whole CT volumes, i.e., all axial slices were processed sequentially without any prior knowledge of whether lesions are present in a slice or not. The statistical analysis was conducted using Python 3.7 (see details in supplementary p. 3).

To evaluate the performance, we used the receiver operating characteristic (ROC) curves^20^ for the lesion detection results (detailed computation process is referred to the “Methods” section). We also computed AUC (i.e., area under the curve) for each ROC curve with 95% CI computed with the DeLong approach^21^. To further evaluate the accuracy of bounding-box areas, we used the metric of mAP (i.e., mean of average precision), which averages the detection precisions under different IoU (i.e., Intersection over Union) rates and is a widely-employed metric for object detectors in image processing^22^. The 95% CI of mAP was computed using the Clopper-Pearson method^23^. In addition, we also analyzed the detection sensitivity and precision at a certain false-positive rate. We chose a value of 0.1 false positive on average per slice, meaning that one false-positive bounding-box prediction is occurred in every ten slices, which is reasonable to be used in clinical practice. We computed the patient-wise variance for the metrics of sensitivity and precision, and their 95% CI of were computed using the z-table^24^.

The *p*-values for detection results between the comparison methods (i.e., Individual-model-1, Individual-model-2, Individual-model-3, model ensemble, joint model), and the federated learning model were computed using the two-sided Student’s *t*-test. The statistical significance was defined as a *p*-value <0.05.

#### Performance on internal and external testing sets with federated learning

**Fig. 2:**
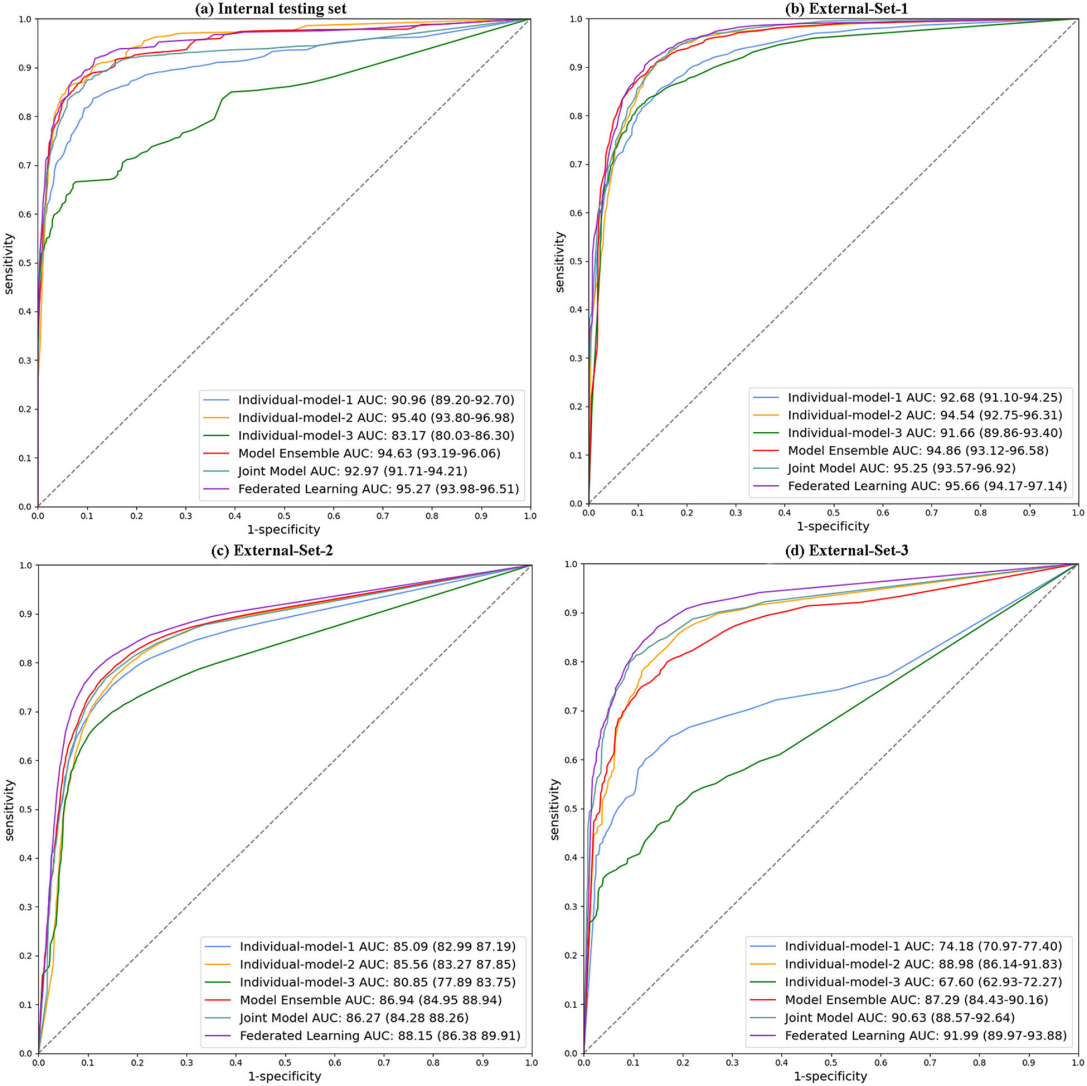
ROC curves for lesion detection on the internal testing set and three external validation sets. Results of six settings are compared, including individual-model-1, individual-model-2, individual-model-3, their model ensemble, their joint training model, and our federated learning model. The results of AUC are presented with 95% CI.

We report the lesion detection results on the internal testing set (15 scans altogether) and three external sets (External-Set-1, External-Set-2, External-Set-3) through comparison with radiologist interpretations. Table 3 lists the mAP value, as well as the detection sensitivity and precision for all the approaches (with *p*-values) on the testing sets. The ROC curves with AUC (with 95% CI) are shown in Fig. 2. Two case studies with External-Set-4 about automated lesion burden estimation with longitudinal scans over time will be discussed in the next section.

**Table 3:** Results of lesion detection in COVID-19 CT on internal testing set and external validation sets.

Models	Internal testing set (Hong Kong)				External cohorts											
					External-Set-1 (Public Dataset)				External-Set-2 (Germany)				External-Set-3 (Mainland China)			
	mAP	Sen	Pre	*p* value	mAP	Sen	Pre	*p* value	mAP	Sen	Pre	*p* value	mAP	Sen	Pre	*p* value
Individual-model-1	83.42 (75.47–91.37)	85.62 (77.33–93.91)	84.4 (77.75–91.05)	0.0002	79.53 (73.33–85.73)	83.54 (78.55–88.53)	86.88 (83.18–90.58)	<0.0001	65.48 (61.76–69.2)	67.36 (64.03–70.69)	90.99 (88.23–93.75)	<0.0001	59.81 (44.45–75.17)	62.78 (47.17–78.39)	84.48 (77.19–91.77)	<0.0001
Individual-model-2	89.98 (82.27–97.69)	92.03 (84.23–99.83)	82.1 (76.16–88.04)	0.1091	84.08 (79.28–88.88)	86.02 (82.12–89.92)	88.95 (85.16–92.74)	0.0002	64.71 (61.06–68.36)	67.17 (63.49–70.85)	90.6 (87.64–93.56)	<0.0001	75.74 (62.17–89.31)	78.76 (66.05–91.47)	87.69 (82.08–93.3)	0.0010
Individual-model-3	72.13 (59.17–85.09)	74.35 (60.7–88.00)	75.21 (63.06–87.36)	<0.0001	79.23 (72.7–85.76)	82.07 (76.58–87.56)	89.26 (85.94–92.58)	0.0009	61.84 (56.95–66.73)	63.41 (59.02–67.8)	90.83 (87.33–94.33)	<0.0001	51.28 (35.25–67.31)	53.19 (37.37–69.01)	78.05 (67.59–88.51)	<0.0001
Model Ensemble	89.48 (81.36–97.6)	91.76 (83.85–99.67)	83.62 (78.07–89.17)	0.1068	84.97 (80.17–89.77)	87.83 (83.93–91.73)	88.96 (85.35–92.57)	0.0419	68.35 (64.98–71.72)	70.31 (67.13–73.49)	91.01 (88.45–93.57)	<0.0001	73.75 (59.27–88.23)	76.31 (62.44–90.18)	85.98 (80.18–91.78)	<0.0001
Federated Learning	91.28 (84.91–97.65)	93.54 (87.33–99.75)	84.21 (78.61–89.81)	–	87.83 (84.18–91.48)	90.35 (87.46–93.24)	88.56 (84.2–92.92)	–	71.48 (68.69–74.27)	73.31 (70.44–76.18)	91.93 (89.48–94.38)	–	83.04 (72.5–93.58)	85.06 (74.82–95.3)	87.94 (83.24–92.64)	–

Our federated learning model is compared with five other different approaches using the single-site, ensemble, and joint training AI models. The table presents the detection mAP (mean Average Precision), and the sensitivity (Sen) and precision (Pre) in percentage at 0.1 false-positive rate. The numbers are presented as mean (95% CIs). For all the testing cohorts, six models are compared, including three individual site models, their model ensemble, their joint training model, and our federated learning model. The *p*-value of each comparison method between our federated model is presented, where *p*-value <0.05 denotes statistical significance.

We first present the results of internal validation. Among three individual models, the Individual-model-2 trained with the largest single-site database (with 9398 lesions) obtained a higher AUC of 95.40% (95% CI 93.80–96.98) than the other two individual models, with the mAP of 89.98% (82.27–97.69), sensitivity of 92.03% (84.23–99.83), and precision of 82.1% (76.16–88.04). The ensemble of three individual models showed a relatively lower AUC 94.63% (93.19–96.06), compared with Individual-model-2. The joint model trained with all internal datasets performs slightly lower than model ensemble, with the AUC of 92.97% (91.71–94.21). Using multicenter data for model training, the federated learning model obtained comparable AUC of 95.27% (93.98–96.51) as Individual-model-2, with slightly higher mAP of 91.28% (84.91–97.65), sensitivity of 93.54% (87.33–99.75), and precision of 84.21% (78.61–89.81). The federated learning model also outperformed the model ensemble method consistently across all the metrics.

**Fig. 3:**
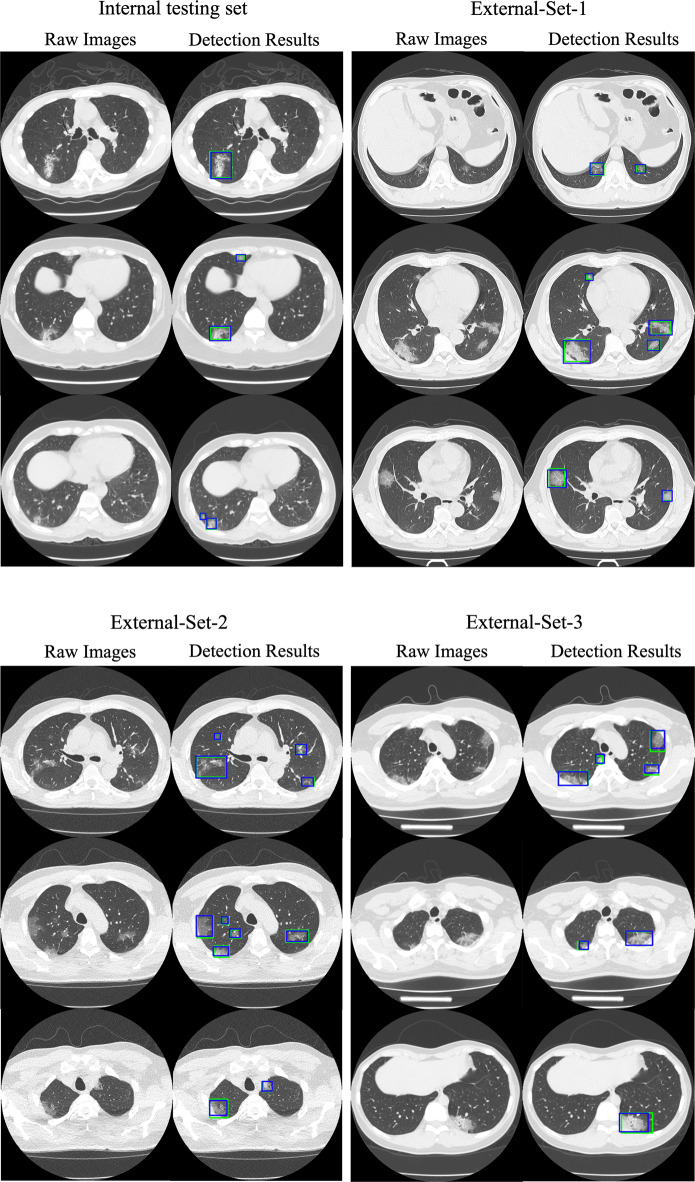
Qualitative results of lesion detection in COVID-19 CT images using federated learning model on the internal testing set and three external sets. The raw images are shown in par with the detection results indicated by bounding boxes. The blue and green boxes denote the model predictions and manual reference lesion regions, respectively.

Next, for generalization performance on unseen external cohorts, on External-Set-1, the best performance was achieved by the federated learning model with AUC of 95.66% (95% CI 94.17–97.14) and mAP of 87.83% (84.18–91.48), among all six methods. For External-Set-2 from Germany with expectable differences in population demographics compared to the training cohorts, the model generalization generally decreased for all models compared with their performance on External-Set-1. The highest performance on this set was also achieved by the federated learning model, with AUC of 88.15% (86.38–89.91), mAP of 71.48% (68.69–74.27), sensitivity of 73.31% (70.44–76.18), and precision of 91.93% (89.48–94.38). The second best results were obtained by the ensemble model through aggregating predictions from all three individual models. External-Set-3 showed similar results, with the federated learning model attaining the highest AUC of 91.99% (89.97–93.88) among all models. Statistical analysis between our federated learning model with other five models revealed a *p*-value <0.05 across all external sets. Figure 3 shows qualitative detection results from our federated learning model on the internal and three external validation sets, illustrating the visual agreement between the predicted and manual reference bounding boxes.

**Fig. 4:**
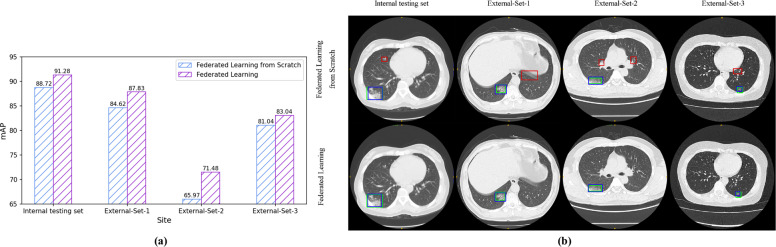
Ablation experiments on federated learning method with and without transfer learning from model pretrained on DeepLesion dataset. Left bar chart shows the performance measured in mAP (mean Average Precision) of two different training strategies, federated learning outperforms federated learning from scratch over all datasets. Right part visualizes the predictions from two methods. The blue, green, and red bounding boxes denote model predictions, manual reference lesion regions, and false-positive predictions, respectively. Transfer learning from pretrained model is helpful to reduce false-positive predictions.

We further conducted ablation study to analyze the effect of transfer learning in our method. Specifically, we trained the federated model in two different learning strategies, i.e., training with model initialized from our previous DeepLesion model^18^ and training from scratch, with results shown in Fig. 4. Compared with training from scratch, the transfer learning model increased mAP by 2.56% on internal testing set and 3.31%, 5.51%, 2.00% on three external sets, respectively. In addition, we visually observed that transfer learning from a large-scale dataset was helpful to reduce the false-positive predictions for lesion detection.

#### Performance on case studies with longitudinal scans for lesion burden estimation

**Fig. 5:**
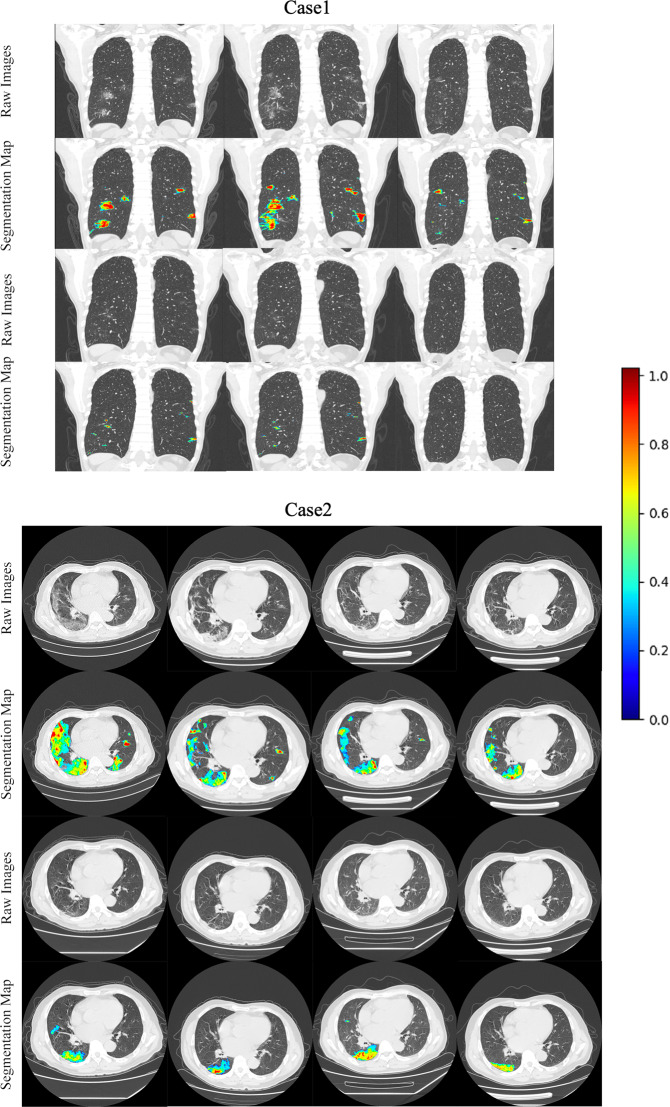
Two case studies with external longitudinal CT scans relying on dense scoremaps of lesion regions for lesion burden estimation. The raw images are shown accompanied with dense prediction scoremaps with the probability color bar. The CT images are chronologically ordered from left to right, top to down, in accordance to the scanning date as shown in the following Fig. 6.

**Fig. 6:**
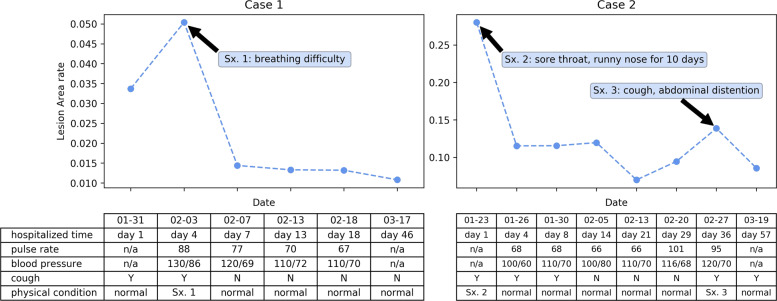
Automated estimation of lesion burden change for longitudinal scans of hospitalized patients. Line curve shows the change of estimated lesion burden under different scanning dates for the two external cases collected in Hubei, China. The table shows the corresponding symptom recorded at each scanning date. Sx = symptom; Y = Yes; N = No.

The potential clinical applicability of our AI model was demonstrated by estimating the lesion burden for monitoring hospitalized patients. This test was done on External-Set-4 with two case studies analyzing the longitudinal scans and clinical symptoms reports. Our detection model can provide lesion segmentation masks without additional supervision by benefiting from transfer learning (details in “Methods” section), and the predicted dense score-map for each scan of the two cases are shown in Fig. 5. We computed the lesion burden as the ratio of lesion segmentation area to whole lung area and observed some correlation of automated estimated lesion burden with clinical symptoms of the patients, i.e., a rise of estimated lesion burden was accompanied with relatively severe clinical symptoms. Figure 6 shows the estimated change of lesion burden in curves across successive time-series CT scans during patients’ hospitalized periods, with their main symptoms listed below as a demonstration. For Case-1 (57 years old, male), the patient’s condition worsened upon admission to hospital (accordingly lesion burden increased from on day-4), and afterward gradually relieved in remaining time (accordingly lesion burden decreased after the peak, and to a very low rate at the last CT before discharge). Such imaging findings were consistent with the patient’s symptoms, showing cough and breathing difficulty along with fast pulse rate and high blood pressure on day-4. These indicators returned to normal level and symptoms alleviated in follow-up, reflecting a trajectory of recovery. For Case-2 (63 years old, male), the estimated lesion burden decreased from the peak at the first scan, and slightly relapsed in the middle period, then recovered on day-57. For the symptoms, the patient reported mild to moderate physical symptoms (sore throat and running nose for 10 days) upon admission to hospital (consistent with the lesion burden peak on day-1), and showed high pulse rate with some physical symptoms during the period from day-29 to day-36 (according to lesion burden fluctuation). Studying the relation between automated lesion burden estimation and clinical symptoms may be important for better understanding this disease, as the clinicians could be objectively informed on whether the patient’s condition is recovering or deteriorating with such automated lesion burden estimation, which may support treatment planning to arrange necessary medical service, especially given that manual labeling of the lesions would be too laborious to extract such an image-derived parameter like lesion burden.

## Discussion

In this study, we demonstrate the feasibility of federated learning to combine COVID-19 data across participating centers in a patient privacy-protecting manner. This decentralized training strategy may be a key enabler of scalable AI-based technology during a pandemic when there is no time to set up complicated data-sharing agreements across institutions or even countries. The use of federated learning has been recently shown in other fields such as edge computing (e.g., digital devices), however, the medical imaging scenario is more complicated and entails unique challenges (e.g., high-dimensional data, imbalanced cohort sizes) which exerts unexplored influences on currently used federated learning techniques. To our knowledge, this is a prior work to demonstrate the feasibility and effectiveness of federated learning for COVID-19 image analysis, where collaborative effort is especially valuable at the time of global crisis. Our experimental observations have shown that federated learning improved generalization performance over all single-site models and their ensemble, reflecting successful decentralized optimization with diverse distributions of training data.

We show in a proof-of-concept that a CNN-based federated deep learning model can be used for accurately detecting chest CT abnormalities in COVID-19 patients. Importantly, the AI model trained on Hong Kong cohorts showed high performance not only on internal testing cases, but also on external, unseen, independent datasets collected from hospitals in Asia and Europe. The scanner brands, imaging protocols, and patient populations varied across these multi-national centers, and the severity of COVID-19 pneumonia differed across the patients involved in the study (see details in supplementary p. 2). The data diversity in this multicenter study demonstrates the feasibility of building robust and generalizable AI tools for combating COVID-19 through image analysis in heterogeneous clinical environments. On average, our model took around 40 ms to test one CT volume, showing potential to support real-time use in practice.

This study hypothesized that multicenter training could enhance the generalizability of AI models, which has previously been demonstrated in other medical imaging scenarios^25–27^. In our experiments, it was first observed that the Individual-model-2 (trained on 4146 CT slices) achieved superior performance over the other two single-site models (trained on 958 and 660 slices) on all three external testing sites. This revealed that larger training databases (even from the same site) could improve model performance on unseen datasets. Moreover, upon merging all three internal sites for training, despite two sites contributing fewer cases, the test accuracy could be further improved. To some extent, this could reflect that, in addition to increased data scale, richer data diversity associated with imaging scanners and protocols may be equally important for reducing model bias thus improving generalizability. In this sense, collaboration across multiple clinical centers is an essential component to pave the way for developing AI systems for wide-spread deployment, especially when faced with COVID-19 pandemic where multinational efforts are crucial.

Although this study recruited patients from seven clinical centers from different regions, the number of patients from each participating center was relatively small. The centers which were heavily involved in managing the pandemic received more cases than other hospitals, resulting in an imbalance between sites. To a certain extent, this reflected the practical situation that COVID-19 patients were generally too scarce for most single sites to train individual in-house AI models. Therefore, multicenter studies with collaborative efforts in data combination are important and valuable to handle the long-tail distribution of COVID-19 patients. Our future work will further include more patients and centers in federated learning. Nevertheless, it is worth noting that, in the current proof-of-concept study, the relatively small numbers of patients did not impede the development of a deep learning model, because the numbers of individual lesions identified from all CT volumes were fairly large (14,095 overall in our datasets).

The model generalized less well on the German cohort, compared with other external cohorts. A reason may be the patient populations coming from different demographics. In addition, the lesion annotation procedures differed between different clinical sites causing what is known in machine learning as concept shift where the manual annotations in this cohort were not directly compatible with training data. For example, the training data annotated ground-glass opacification and consolidation, while the German cohort contained a few pleural effusion lesions, which are atypically seen in COVID-19, with relatively low contrast against normal lung tissue. We excluded 15 cases (with very mild lesions which could hardly be seen in the lung window), 5 cases (with severe diffuse lesions which were not suitable to be processed as a detection task predicting lesion bounding boxes), and 1 case (with no CT finding). Supplementary Fig. 1 in the supplementary (p. 4) shows typical images from patients excluded from German cohort. By doing this, 35 cases remained with the model obtaining AUC of 88.15% (95% CI 86.38–89.91). If tested on all German data of 56 cases, the model obtained AUC of 77.15% (95% CI 72.84–81.47). We envision the CT abnormality detection tool to be used alongside the standard visual assessment by expert radiologists. In that way, the AI tool supports the expert by providing quantitative measurements during clinical decision-making. At the same time, the expert in the loop acts as a safeguard against erroneous predictions such as false positives in non-suspicious scans. It is also worth noting that concept shift as present in the German cohort can be avoided in future multicenter studies that may use federated learning when all sites follow a standard operating procedure. Due to the opportunistic nature of this study where data from multiple sites was included and each cohort had been collected independently, this was not possible. Despite these limitations, we believe that our multinational validation confirms the potential for our approach while highlighting the real-world challenges in such studies.

In conclusion, the CNN-based AI model trained using a privacy-protecting federated learning approach is effective in detecting CT abnormalities in COVID-19 patients. The wide generalizability to regional and international external cohorts, benefited from including diverse datasets, shows the promise of AI providing low-cost and scalable tools for lesion burden estimation to support clinical disease management.

## Methods

### Ethical approvals obtained by internal cohorts

In this multicenter study, the internal datasets were collected from three local hospitals in Hong Kong, i.e., Prince Wales Hospital (PWH), Princess Margaret Hospital (PMH), and Tuen Mun Hospital (TMH). The ethical approvals were obtained in accordance with all relevant laws and regulations for each recruiting hospital, i.e., PWH approved by the Joint Chinese University of Hong Kong - New Territories East Cluster Clinical Research Ethics Committee, PMH approved by the Kowloon West Cluster Research Ethics Committee, TMH approved by the New Territory West Cluster Research Ethics Committee.

### Federated learning process

To protect data privacy during model training, we studied the feasibility of federated learning on three local hospitals, with each individual center representing a node. Data sharing across the sites was not required, while the model benefitted from the generalizability enabled by multicenter learning with the inclusion of diverse data sources. More specifically, such a decentralized scheme trained individual models on local nodes and exchanged the network parameters to update a global model stored at the central server at a certain frequency (i.e., every training epoch). In each iteration of the federated update, the central server first aggregated all the local models and used them to update the global model parameters using the Federated Averaging (FedAvg) algorithm^28^. The updated global model was generated by using a weighted average of the parameters from all the local models, weighted proportionally to the sample size on each node, which is provided by local node to the global server. Next, the central server distributed the updated global model to the local nodes, then each node continued to perform local optimizations based on the updated global model with its local data. After an epoch, each node sent back the updated parameters to the central server for the next federated learning iteration. This process was repeated until the global model converged.

Formally, assume that there are *K* (*K* = 3 in our setting) hospitals for collaborative training, with *n*_*k*_ as the number of data points in each hospital *k*. At the beginning of one federated training round *t*, the central server first sends the global model with parameters *w*_*t*_ to all local hospitals. Each hospital *k* then optimizes the received model locally with its own dataset for *E* epochs (*E* = 1 in our implementation), and then sends the model update ∇*w*_*t*_^*k*^ back to server. Once receiving the updates from all local hospitals, the server averaged these updates with weights in proportion to the sizes of local dataset to refurbish the global models as with a learning rate. Such a process repeats until the global model converges. Note that for each hospital participating federated learning, the sample size of its local dataset is given to the central server for aggregating the local parameters with weighted average to update the global model.

### Network architecture and transfer learning

Transfer learning from the public large-scale DeepLesion^19^ CT lesion dataset was effective to handle the data insufficiency issue for developing the deep learning detector with COVID-19 data in this study. The dataset is currently the most comprehensive open-source data for CT lesions, that includes 32,120 axial CT slices (from body parts of liver, lung, mediastinum, kidney, pelvis, bone, abdomen, and soft tissue) from 10,594 studies of 4427 unique patients. For network architecture of our model, we used our previously developed improved version of RetinaNet^18^, a 2D CNN with medical-domain customized design for object detection based on the original RetinaNet backbone^29^. The model input consisted of three adjacent CT axial slices. Our model was initially pretrained on the DeepLesion dataset (relying on our previous work^18^), then fine-tuned with internal COVID-19 training images. In our previous work, we utilized RECIST diameters provided in DeepLesion dataset as weak labels to generate pseudo-masks for learning an auxiliary branch for lesion segmentation (i.e., predicting the dense masks of lesions). This segmentation branch with associated parameters was learned in the pre-training step, while kept frozen in the fine-tuning process without further update, as lesion segmentation annotation for our dataset was unavailable. We found that this auxiliary segmentation branch could still output lesion segmentation masks with acceptable quality at testing (as shown in Fig. 5), thanks to transfer learning from a closely-related domain. This supported that the model pretrained on various other types of lesions in CT could capture general patterns of abnormalities, which is also applied for the novel disease of COVID-19 to some extent.

### Pre-processing, model training, and post-processing

For pre-processing, we clipped the Hounsfield units (HU) for each volume before rescaling their intensities to [−1.0, 1.0]. From experimental observations, we found that instance-level normalization to zero mean, unit variance helped improve generalizability, i.e., normalizing every single volume with its individual statistics rather than using the dataset global statistics. After normalization, three adjacent slices were combined as the input for CNN-based models.

In each round of federated training, every local client optimized their model for one epoch using the individual dataset. All local clients used Adam optimizer with hyper-parameter as learning rate of 1e−4, beta1 of 0.9, beta2 of 0.999, and epsilon of 1e−7. We applied data augmentation schemes including random horizontal and vertical flipping with 50% probability; random clockwise rotation with amplitude ranging from −0.1 to 0.1; random horizontal/vertical translation with −0.1 to 0.1 of input image length/width; and random shearing and scaling with variation of −0.1 to 0.1. The data augmentations were performed with Numpy and TensorFlow libraries through processing the data arrays. A small amount of training data was held out at each node to determine the model convergence. If the global model performance on local validation data was not increased for five successive federated rounds, we considered that the training was converged and the federated learning was stopped. The deep convolutional networks were trained with one NVIDIA TitanXp GPU.

For the post-processing, we used non-maximum suppression^30^, a well-established method in image processing to retain a single entity out of overlapping entities. In our case, we adopted it to extract the bounding boxes with the highest predicted probability from a series of overlapped bounding boxes. Specifically, given a number of predicted bounding boxes from an image, we remove bounding boxes with probability lower than a threshold. With the remaining bounding boxes, we repeatedly keep the bounding box with the highest probability and discard remaining boxes with IoU higher than 0.5 with this selected one. We also applied an existing open-source lung segmentation AI model^31^, to remove the false-positive detections which fell outside the lung region.

### Method to compute ROC curves in statistical analysis

Given an input CT volume, each axial slice was sequentially tested with the AI models. The outputs of these detection models were a set of bounding-box predictions (a.k.a. proposals), each of which carried a score to indicate the probability of current prediction being a lesion. We used the prediction score on which a threshold varied to calculate the ROC curves. For each proposal, if its IoU (i.e., Intersection over Union) with any of the manually labeled bounding boxes being higher than 0.5^31,32^ (following the de facto setting in literature), it was identified as a true positive result. On the other hand, for each prediction, if its IoU with all of the labeled bounding boxes being smaller than 0.5, it was identified as a false-positive result. With these true positives and false positives, we computed the sensitivity and specificity pairs to obtain the ROC curves.

### Reporting summary

Further information on research design is available in the Nature Research Reporting Summary linked to this article.

## Supplementary information

Supplementary Information

Reporting Summary

## Data Availability

Raw images of the public dataset are accessed at https://coronacases.org/, and the annotations can be obtained from https://gitee.com/junma11/COVID-19-CT-Seg-Benchmark. Data from Hong Kong will be available after approval by the relevant corresponding authors. Data from Shenzhen, China; Hubei, China; and Munich, Germany, contain confidential information and are not authorized to be shared openly at this stage. Qualified researchers with reasonable requests for access of the data should contact the relevant authors of these institutions. Any data use will be restricted to non-commercial research purposes.

## Code availability

The source code including both training and testing of our networks, with pretrained model and demo data, are available at https://github.com/med-air/FL-COVID.
