# Supplementary material for: Federated deep learning for detecting COVID-19 lung abnormalities in CT: a privacy-preserving multinational validation study
Source: NPJ Digit Med. 2021 Mar 29;4:60. doi: 10.1038/s41746-021-00431-6 (PMC8007806; doi:10.1038/s41746-021-00431-6)
Supplement: Supplementary file 1 — Supplementary Information [file 41746_2021_431_MOESM1_ESM.pdf]

npj | digital medicine

Supplementary information

Supplement to: **Federated deep learning for detecting COVID-19 lung abnormalities in CT: A privacy-preserving multinational validation study**, by Qi Dou, Tiffany Y So, Meirui Jiang, Quande Liu, Varut Vardhanabhuti, Georgios Kaissis, Zeju Li, Weixin Si, Heather HC Lee, Kevin Yu, Zuxin Feng, Li Dong, Egon Burian, Friederike Jungmann, Rickmer Braren, Marcus Makowski, Bernhard Kainz, Daniel Rueckert, Ben Glocker, Simon CH Yu, Pheng Ann Heng.

Index of Supplementary

Detailed annotation process of datasets. …………………………………….……….….………...2

Variations in patient severity. …………………………………………………………………...2

Statistical analysis details. ……………………………………………………………….…...2

Supplementary Figure 1: visualization of typical samples excluded from Germany cohort..3

Refrences. ……………………………………………………….………………….…4

Detailed annotation process on internal cohorts

The regions of ground-glass opacification and consolidations, which are the two main signs of COVID-19 assessed on CT images, were manually annotated with bounding boxes based on a slice-by-slice labelling scheme using ITK-SNAP (version 3.8.0-beta) under the lung window setting (width, 1600 HU, level, −600 HU). Our annotation process involved one radiologist (TS) with 8 years of clinical experience, and one trained scientist (DQ) with 7-year research experience in computational medical image field. These two annotators independently reviewed all the cases from three local cohorts, and their annotation consistency in terms of kappa coefficient was 91·27% on Internal-Set-1, 89·81% on Internal-Set-2, and 91·17% on Internal-Set-3, which presented almost perfect agreement [1]. The consensus labels from these two annotators were directly accepted. A third expert radiologist (VV) served as a third-party verification role for any discrepancy from the first two reviewers. For each internal cohort, the patients were randomly divided into around 80% to be used as the training subset, and the remaining 20% for the testing subset.

Variations in patient severity

Our patients were heterogeneous in the stage of their disease, i.e., the distribution of severity between CT and symptom onset. This reflected the real-world scenario as patients might be admitted to hospitals at different time frames or stages. As a result, the recruited patients broadly showed different levels of disease severity, which brought about significant variations in terms of imaging findings. Specifically, our three internal cohorts covered 61·3% mild cases, 22·7% moderate cases and 16·0% severe cases based on our radiologist interpretations. The experiments merged all available patients for training and testing regardless of their disease severity. We qualitatively observed that our AI system performed less well in ground-glass lesions from mild cases, the predictions covered the core area of lesion with periphery undetected, compared with consolidative opacities which were common in moderate or relatively severe cases.

Statistical analysis details

Package usage for statistical analysis. All statistical analysis was done using the Python language (version 3·7·7), with the following packages: numpy (version 1·17·5), scipy (version 1·4·1), scikit-learn (version 0·22·1). Images were loaded using SimpleITK (version 1·2·4) and all visualizations were performed with matplotlib (version 3·2·1).

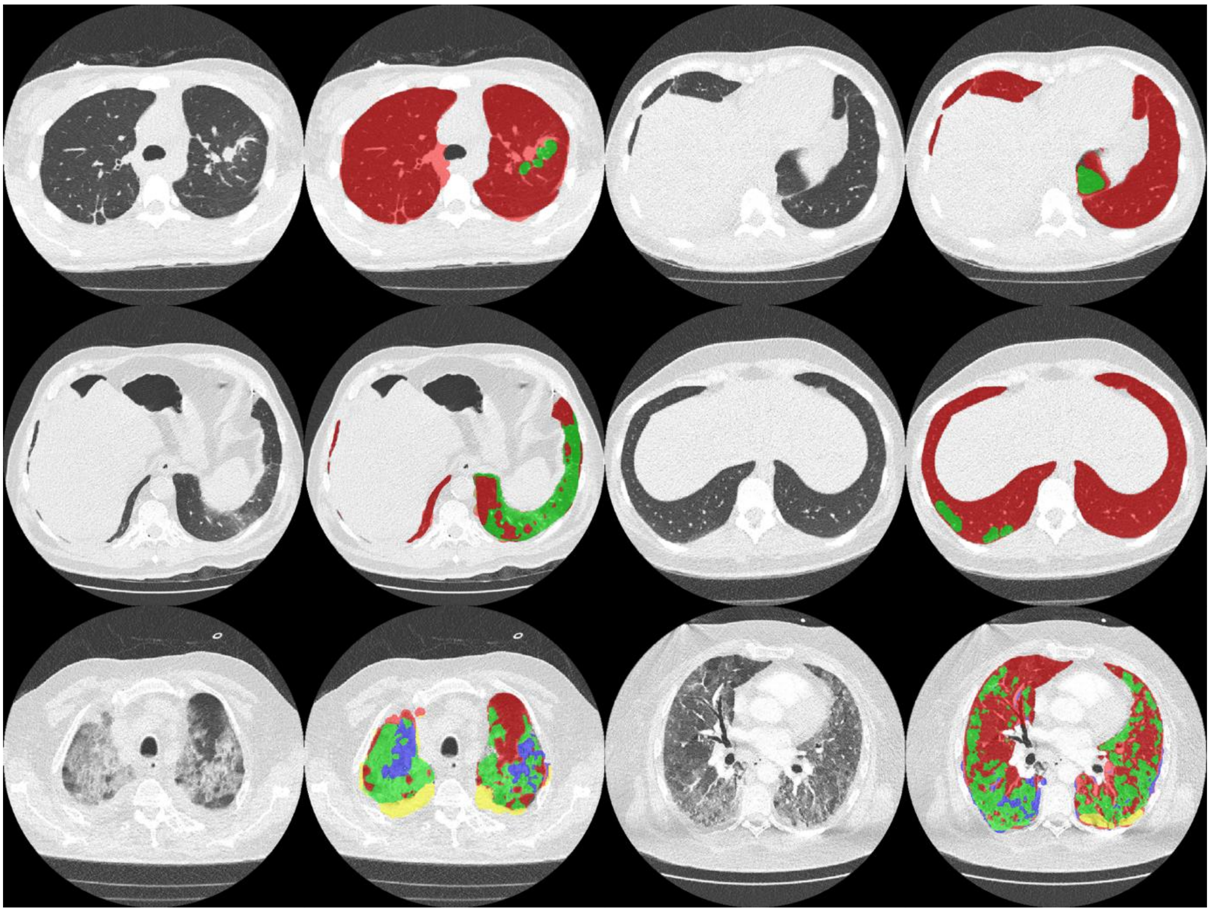

Supplementary Figure 1: Visualization of typical samples excluded from German cohort.

The raw images are shown in par with their corresponding annotations. The red, blue, yellow and green labels represent the whole lung, ground glass opacification, consolidation and pleural effusion, respectively. The first two rows show instances of mild changes with concept shift, and the last row displays examples of severe cases with diffuse changes.

Reference

1. Kundel H L, Polansky M. Measurement of observer agreement[J]. Radiology, 2003, 228(2): 303-308.
