# Supplementary material for: Federated deep learning for detecting COVID-19 lung abnormalities in CT: a privacy-preserving multinational validation study
Source: NPJ Digit Med. 2021 Mar 29;4:60. doi: 10.1038/s41746-021-00431-6 (PMC8007806; doi:10.1038/s41746-021-00431-6)
Supplement: Supplementary file 2 — Reporting Summary [file 41746_2021_431_MOESM2_ESM.pdf]

nature research

Corresponding author(s): Qi Dou, Ben Glocker, Simon CH Yu, Pheng Ann Heng

Last updated by author(s): Feb 23, 2021

Reporting Summary

Nature Research wishes to improve the reproducibility of the work that we publish. This form provides structure for consistency and transparency in reporting. For further information on Nature Research policies, see our Editorial Policies and the Editorial Policy Checklist.

Statistics

For all statistical analyses, confirm that the following items are present in the figure legend, table legend, main text, or Methods section.

n/a	Confirmed	
☐	☒	The exact sample size (n) for each experimental group/condition, given as a discrete number and unit of measurement
☐	☒	A statement on whether measurements were taken from distinct samples or whether the same sample was measured repeatedly
☐	☒	The statistical test(s) used AND whether they are one- or two-sided<br>*Only common tests should be described solely by name; describe more complex techniques in the Methods section.*
☐	☒	A description of all covariates tested
☐	☒	A description of any assumptions or corrections, such as tests of normality and adjustment for multiple comparisons
☐	☒	A full description of the statistical parameters including central tendency (e.g. means) or other basic estimates (e.g. regression coefficient) AND variation (e.g. standard deviation) or associated estimates of uncertainty (e.g. confidence intervals)
☐	☒	For null hypothesis testing, the test statistic (e.g. F, t, r) with confidence intervals, effect sizes, degrees of freedom and P value noted<br>*Give P values as exact values whenever suitable.*
☒	☐	For Bayesian analysis, information on the choice of priors and Markov chain Monte Carlo settings
☒	☐	For hierarchical and complex designs, identification of the appropriate level for tests and full reporting of outcomes
☒	☐	Estimates of effect sizes (e.g. Cohen's d, Pearson's r), indicating how they were calculated

Our web collection on statistics for biologists contains articles on many of the points above.

Software and code

Policy information about availability of computer code

Data collection	no software was used to collect the data in this study
Data analysis	Python language (version 3·7·7), numpy (version 1·17·5), scipy (version 1·4·1), scikit-learn (version 0·22·1), SimpleITK (version 1·2·4), matplotlib (version 3·2·1), ITK-SNAP (version 3.8.0-beta).

For manuscripts utilizing custom algorithms or software that are central to the research but not yet described in published literature, software must be made available to editors and reviewers. We strongly encourage code deposition in a community repository (e.g. GitHub). See the Nature Research guidelines for submitting code & software for further information.

Data

Policy information about availability of data

All manuscripts must include a data availability statement. This statement should provide the following information, where applicable:

- Accession codes, unique identifiers, or web links for publicly available datasets
- A list of figures that have associated raw data
- A description of any restrictions on data availability

Raw images of the public dataset are accessed at https://coronacases.org/, and the annotations can be obtained from https://gitee.com/junma11/COVID-19-CT-Seg-Benchmark. Data from Hong Kong will be available after approval by the corresponding authors. Data from Shenzhen, China; Hubei, China; and Munich, Germany contain confidential information and are not authorized to be shared openly at this stage. Qualified researchers with reasonable requests for access of the data should contact the relevant authors of these institutions. Any data use will be restricted to noncommercial research purposes.

Field-specific reporting

Please select the one below that is the best fit for your research. If you are not sure, read the appropriate sections before making your selection.

☒ Life sciences ☐ Behavioural & social sciences ☐ Ecological, evolutionary & environmental sciences

For a reference copy of the document with all sections, see nature.com/documents/nr-reporting-summary-flat.pdf

Life sciences study design

All studies must disclose on these points even when the disclosure is negative.

Sample size	The sample size is 132 patients from 7 centers with 41,966 independent lesions annotated. Our experimental results could validate the sample size is sufficient for this study.
Data exclusions	No data were excluded from the analyses.
Replication	We have released the code and checked the reproducibility of the experimental findings.
Randomization	Training and testing samples were randomly divided in the deep federated learning study.
Blinding	Blinding was not relevant to this study on artificial intelligence of medical image analysis.

Reporting for specific materials, systems and methods

We require information from authors about some types of materials, experimental systems and methods used in many studies. Here, indicate whether each material, system or method listed is relevant to your study. If you are not sure if a list item applies to your research, read the appropriate section before selecting a response.

Materials & experimental systems

n/a	Involved in the study
☒	☐ Antibodies
☒	☐ Eukaryotic cell lines
☒	☐ Palaeontology and archaeology
☒	☐ Animals and other organisms
☒	☐ Human research participants
☒	☐ Clinical data
☒	☐ Dual use research of concern

Methods

n/a	Involved in the study
☒	☐ ChIP-seq
☒	☐ Flow cytometry
☒	☐ MRI-based neuroimaging
